# Metrics Toolkit: an online evidence-based resource for navigating the research metrics landscape

**DOI:** 10.5195/jmla.2018.526

**Published:** 2018-10-01

**Authors:** Robin Champieux, Heather Coates, Stacy Konkiel, Karen Gutzman

**Affiliations:** Research Engagement & Open Science Librarian, OHSU Library, Oregon Health & Sciences University (OHSU), Portland, OR 97239-3098; Digital Scholarship and Data Management Librarian, IUPUI University Library Center for Digital Scholarship, Indiana University Purdue University Indianapolis (IUPUI), Indianapolis, IN 46202; Director of Research and Education, Altmetric, WeWork-Kings Place, 7th Floor, 90 York Way, London, N1 9AG, United Kingdom; Impact and Evaluation Librarian, Galter Health Sciences Library & Learning Center, Northwestern University, Chicago, IL 60611

## Abstract

While research metrics may seem well established in the scholarly landscape, it can be challenging to understand how they should be used and how they are calculated. The Metrics Toolkit is an online evidence-based resource for researchers, librarians, evaluators, and administrators in their work to demonstrate or assess the impact of research.

The Metrics Toolkit is available online and allows users to browse and explore metrics or choose specific metrics to learn more about. The Metrics Toolkit has filters to guide the user in finding appropriate metrics based on the type of impact, the research object, or the discipline being assessed. Currently, the Metrics Toolkit includes a mix of citation-based metrics and altmetrics.

Each metric in the Metrics Toolkit has a clear definition, explanation of how the metric is calculated, descriptions of its limitations, suggestions of inappropriate use cases, and notes on how the metric is best used. There are also helpful use cases for how metrics can be used in curricula vitae (CVs), promotion dossiers, or grant applications.

Librarians can use the knowledge they gain from the toolkit for many purposes, including:

- assisting faculty with finding metrics to use in their biographical statements, CVs, biosketches, or promotion and tenure packets
- guiding administrators in selecting appropriate metrics that align with the institutional values for assessment projects
- helping researchers identify metrics to enhance their grant applications and progress reports to funders
- providing information to departments on useful metrics for academic program reviews
- identifying highly disseminated or impactful publications by faculty and staff

The Metrics Toolkit was developed by Robin Champieux, Heather Coates, and Stacy Konkiel, using funding from the 2016 Force11 PitchIT! Innovation grant, and was formally launched in January 2018. A year before the tool was launched, the team created an advisory board of librarians, researchers, and evaluators to offer guidance and feedback on the Metrics Toolkit through phone meetings and email. The team developed a project outline that included milestones and deliverables, all of which were made available on Google Drive. They decided on the initial scope of metrics for the toolkit based on input from the advisory board and informal interviews with faculty at their institutions on their use of metrics for research evaluation. Once the initial metrics were chosen, the team completed a literature search on the background, use cases, data sources, and limitations for each metric. They compiled the information and relevant citations into a single document and formulated a template for the metrics that would be displayed on a website.

After some exploration into website templates, the team decided to develop the tool using WordPress, which offers many customizable templates that are easy to use and freely available. The team developed most of the website using an existing WordPress template, though they worked with a freelancer to implement some customization to the look and feel of the user interface. The website is hosted by Altmetric.com, and the toolkit receives ongoing support from the Oregon Health & Science University, Indiana University Purdue University Indianapolis (IUPUI), and Altmetric.com. Additionally, the project maintains a GitHub repository, where content for each metric is maintained and community members can suggest enhancements.

The Metrics Toolkit has been promoted via social media (on Twitter: @Metrics_Toolkit) and was presented at the 2017 Force11 Conference. The team identified several librarians and researchers to help champion the project through social media, blogs, and library guides. The mission, team members, metrics schema description, and a road map for future enhancements are available online. Future enhancements include adding coauthorship metrics, tagging metrics with disciplinary-appropriate information, and exploring metrics for alternative research outputs.

